# Retrospect of the Medical Sciences

**Published:** 1843-04-15

**Authors:** 


*On the use of the Liquor of Hydriodate of Arsenic and Mercury in Cutaneous and Uterine Affections.* By Isaac E. Taylor, M. D., of New York.

In volume 16th of the Dublin Medical Journal, M. Donovan recommends to the profession, a new chemical combination entitled "*Liquor Hydriodatis Arsenici et Hydrargyri.*" In the last eighteen months I have prescribed this preparation in a number of cutaneous diseases, and I feel happy in being able to testify that it produces a more marked and prompt effect than the various remedies usually resorted to in those intractable varieties, Lupus, (its different forms,) Rupia, Psoriasis, Secondary Venereal, &c.

Mr. Donovan's formula for the "*Liquor Hydriodatis Arsenici et Hydrargyri,*" is the following:

Triturate 6.08 grains of finely levigated metallic arsenic, 15.38 grains of mercury, and 50 grains of iodine, with one drachm measure of alcohol, until the mass becomes dry, and from being deep brown has become pale red. Pour on eight ounces of distilled water; and after trituration for a few moments, transfer the whole to a flask; add half a drachm of hydriodic acid, prepared by the acidification of two grains of iodine, and boil for a few moments. Where the solution is cold, if there be any deficiency of the original eight ounces, make it up exactly to that measure with distilled water. Finally filter.

His theory of this process is the following. By the long continued trituration of arsenic, mercury, iodine and alcohol, the metals are converted into iodides, which combine. The mass, by solution in water, is converted into an hydriodate of arsenic and mercury. The quantities of the two metals are so adjusted, that, when converted into protoxides by decomposition of a portion of the water in which they are dissolved, there will be eight grains of protoxide of arsenic, and sixteen of protoxide of mercury. The quantity of water is such that each drachm measure of the solution will contain exactly one-eighth of a grain of protoxide of arsenic, and one-fourth of a grain of protoxide of mercury. Mr. Donovan conceives that the quantity of mercury ought to be double that of the arsenic, in order to insure a slow and moderate, yet adequate mercurial action along with the proper effect of the arsenic.

Of this liquor hydriodatis arsenici et hydrargyri, each drachm measure consists of:

Water, one drachm.
Protoxide of arsenic, one-eighth of a grain.
Protoxide of mercury, one-fourth of a grain.
Iodine (converted into hydriodic acid) four-fifths of a grain.

The colour of this solution, Mr. D. states is yellow, with a pale tinge of green: its taste is slightly styptic. It cannot be properly conjoined with tincture of opium, or with sulphate, muriate, or acetate of morphia; for all these produce immediate and copious precipitates in it. Hence if opiates are to be used during the exhibition of this arsenico-mercurial liquor they must be taken at different periods of the day. Tincture of ginger produces no bad effect.

Mr. Donovan recommends the following formula:

℞.—Liquoris Hydriodatis arsenici et hydrarg. drachmas duas; aquæ destillatæ uncias tres cum semisse; Syrupi Zingiberis semunciam. Misce—Divide in haustus quatuor. Sumatur unus mane nocteque.

Thus one-sixteenth of a grain of protoxide of arsenic, and one-fourth of a grain of protoxide of mercury would be taken in each dose, along with two-fifths of a grain of iodine, which being in a state of combined hydriodic acid, will be much diminished in energy of medical effect. This is according to Mr. D. the proper dose to begin the exhibition of arsenic with, but it will very soon be necessary, he says, to increase it.

The division into draughts is necessary; first to insure accuracy of the dose, so essential in the case of this active medicine; and next to prevent injury to the ingredients by the use of a metallic spoon as a measure.—*American Journal of the Medical Sciences.* April, 1843.

---

### EXTIRPATION OF A TUMOR INVOLVING THE PAROTID GLAND.

BY BENJAMIN TRAVERS, JUN.

On the 21st of December last I was called upon to remove a large tumor from the side of the face of a married lady in her 29th year, of a leuco-phlegmatic aspect, subject to occasional menorrhagia, dyspeptic, and often ailing, but not apparently strumous. The tumor occupied the parotid space, extending downward into the neck, below and behind the angle of the jaw, and upwards in the direction of the lobe of the ear, which was much raised and projected by its increase. It was flat though prominent, having a smooth surface, generally moveable, but somewhat adherent at the neck, and towards the angle of the jaw.

It was not painful, but perceptibly on the increase, so that deglutition was at times impeded. This tumor had existed twelve months, and, so far as the patient knows, its origin was strictly spontaneous. Mr. Travers was consulted, and the operation had his sanction and superintendence.

The patient was placed in the sitting posture, and the incision extended from behind the ear downwards and forwards towards the angle of the jaw, below and behind which it terminated, a full inch distant from that process. The first stage of the dissection was quickly completed, leaving the superficial investment of the parotid gland exposed in all directions. I then began to raise the growth carefully from before backwards, dividing freely the stylo-maxillary insertion of the fascia, and laying bare the fibres of the masseter muscle. In cutting the fascia an artery was wounded, and secured by ligature. The mass was now much loosened, and protruded considerably, discovering its bulk to be far greater, and its remaining connexions firmer, than was at first suspected. Up to this point nothing had appeared but the healthy though expanded texture of the parotid. On recommencing the dissection on the under side of the growth, there followed upon a slight touch of the knife a sudden protrusion of an apparently steatomatous mass, shelling out from a membranous capsule, and exceeding two inches in its greatest diameter. This was pulled away with the fingers. The collapsed cyst, and the superincumbent gland to which it belonged, remained to be removed. This dissection was the most hazardous and difficult part of the operation, for the pulsations of the carotid artery were clearly visible in the bed of the wound, which was now of considerable depth, and bleeding freely. Much was effected by forcible laceration, aided occasionally by delicate touches with the point of the knife. In this manner the operation was completed. Just before its close a considerable artery was divided near the neck of the jaw. This was secured with some difficulty. I believe it to have been the transversalis faciei, very near its origin.

The following parts were all exposed and visible. The neck of the lower jaw; the whole of its vertical ramus, inclusive of the angle; the masseter muscle in part; the mastoid process, and attachment of the sterno-mastoid muscle. The place of the external carotid was marked by the presence of strong pulsation; but I do not affirm that the proper external coat of the vessel was seen. Its sheath, I believe, formed the boundary of the gap in that direction. The patient was much exhausted by the operation, which lasted about twenty-five minutes. As soon as the circulation had recovered, a full opiate was given, which procured repose rather than much sleep. It is unnecessary to dwell upon the after-management of the case. Hæmorrhage supervened at midnight, owing to the parting of a ligature. The skin was distended by soft clots, which were passing through the wound for many days, being aided in their escape by a poultice. Union progressed rapidly under plaster and a light compress, confined by a roller carried over the vertex and occiput. The last ligature separated kindly within the first fortnight, and there now remains nothing but a linear cicatrix, interrupted by a small chink, through which a little saliva distils during mastication.

There is another consequence of these operations to which this case offers, for the present, no exception, viz., a partial paralysis of the side of the face. The angle of the mouth droops, and the upper eyelid closes imperfectly. Sensation has, on the contrary, been morbidly excited, and the integuments were, for a time, sore and swollen. An inspection of the gland, which is now in the museum of St. Thomas's Hospital, explains the cause of these symptoms. The ramifications of the facial nerve have been in part removed. Experience has shown that this is an unavoidable consequence of all such operations; but from the testimony of authors,* and other considerations, I am induced to think that motion will be eventually regained.

There were present at this operation Mr. Travers, Mr. Wakefield of Battle Bridge, and Mr. Croft of Arthur Street. I believe the general impression to have been, that the parotid gland was removed on the present occasion. Some molecular pieces no doubt still remain, of which the subsequent secretion is an evidence; but that the central mass of the organ was extirpated I entertain no doubt.

Whatever anatomists may be pleased to affirm to the contrary, the possibility of such removal has been confirmed by repeated experience. I will merely allude to the works of Velpeau, Liston, and Warren, amongst others, as furnishing abundant proof of the fact; and I may here refer to a case which occurred in the London Hospital, and which is reported in your journal of the 5th February, 1831. This operation was performed successfully by Mr. Luke, to whom I am indebted for the foregoing reference.—*Lon. Med. Gaz.* Feb. 24, 1843.

---

### REMARKS ON ABNORMAL SOUNDS IN THE EAR.

BY G. KRAMER, M. D., BERLIN.

The occurrence of accidental or abnormal sounds or noises in the ear has been accounted for in three ways. 1. It is said that the sound is caused in the interior of the organ of hearing; 2. That it arises from condensation of air in the cavity of the tympanum; and 3. It depends on tension of the membrana tympani.

Although experience has never demonstrated the existence of aneurismal dilatation of the small vessels of the internal ear, and although the small calibre of the vessels must render such an accident very improbable, yet Itard seems disposed to admit it in certain cases giving rise to the development of certain sounds in the interior of the ear. This, however, is a mere hypothesis. The pulsations of which some plethoric individuals complain, have likewise been explained by distension of the arteries of the ear; but it is much more probable that sounds of this kind are produced in the sinuous canals through which the bloodvessels pass to the brain, and that they are rather felt in the head than heard in the ear. The same remark is applicable to the sounds which attend aneurism of the primary carotid and arch of the aorta, or hypertrophy of the heart.

As to condensation of air in the cavity of the tympanum, it is impossible to conceive how such an effect could be produced. Previous obliteration of the Eustachian tube must coincide with such an accident; but then the retained air would be dilated by the natural heat of the body, instead of being condensed. Now, dilatation of the air in the cavity of the tympanum is not a cause of "singing" in the ear, for this symptom is as often absent as present in cases of obliteration of the Eustachian tube.

The third alleged cause is likewise inadmissible. The membrana tympani is always in a state of tension, in healthy persons free from the noises alluded to; it is of a shining hue, and very convex outwards; I have never seen this membrane more tense than it should be in several hundreds of patients whom I have examined with the greatest care, and I am unacquainted with any symptom indicative of this pretended excess of tension. The only alterations which it presents are changes of texture; we find the membrana tympani thickened, inflamed, destroyed in part or in whole, opaque, rough, covered with vegetations, &c., without being able to trace any connection between these organic lesions and the production of abnormal sounds in the ear.

It has been affirmed that all obscurity in the diagnosis of increased excitability of the auditory nerve, as a cause of abnormal sound, ceases as soon as the symptom becomes connected with deafness. By deafness we are here to understand diminution, and not a complete loss of the power of hearing; for mention is made of deafness which has suddenly ceased on the disappearance of the sounds, while the sudden disappearance of true deafness is a thing absolutely impossible. But deafness, (or rather hardness of hearing,) accompanied by irregular noises in the ear, is not dependent on some affection of the auditory nerve alone. It is accompanied by various diseases of the auditory apparatus, and this so often, that we cannot admit, from the coexistence of deafness with abnormal sounds that the latter depend on increased sensibility of the auditory nerve.

To prove this assertion I have taken from my notebook one thousand cases of diseases of the ear, and I have classed them in two series, according as they were or were not accompanied by accidental sounds; the species of disease was noted in each case after careful examination. The following are the results obtained:

---

*Vide Warren on Tumors.

| | With sounds. | Without sounds. |
|---|---|---|
| Erysipelatous inflammation of the meatus and obstruction by cerumen - - - - - | 72 | 22 |
| Inflammation of the glandular apparatus of meatus - - - | 11 | 10 |
| Inflammation of its cellular tissue - - - - - - - - | 8 | 0 |
| Inflammation of its periosteum | 1 | 3 |
| Acute inflammation of membrana tympani - - - - | 3 | 1 |
| Chronic inflammation of membrana tympani - - - - | 95 | 81 |
| Congestion of Eustachian tube | 40 | 44 |
| Contraction of Eustachian tube | 11 | 8 |
| Obliteration of Eustachian tube | 2 | 0 |
| Inflammation of cellular tissue of cavity of tympanum - - | 2 | 1 |
| | 245 | 170 |
| Increased sensibility of auditory nerve - - - - - - | 462 | 123 |
| | 707 | 293 |
| | 1000 | |

In all these cases the power of hearing was more or less diminished. In 707 of them the deafness was accompanied by abnormal sounds; and we should, therefore, (if the doctrine alluded to be true,) admit increased excitability of the auditory nerve; but this was absent in 245 of the 707 cases. The coexistence of deafness and "singing" in the ears is not, therefore, a certain sign of exalted excitability of the acoustic nerve, since it does not occur in 245 out of 707 cases; while of 585 cases, in which this increased excitability did exist, 123 were unattended with any trace of abnormal sounds in the ear. The mistake of writers on this subject depends on their explanation of abnormal sounds considered as a symptom of disease.

From the numerous observations which I have made, it appears to me that the duration and interruption of these sounds, their intensity, weakness, and infinite degrees of diversity are in no way connected with any particular affection of the auditory apparatus. The same individual may labour under the same form of disease in both ears, yet he shall hear the sounds on one side and be free from them on the other. Hence we must conclude that the cause of these sounds remains enveloped in the greatest obscurity, and that we cannot attach to them the slightest importance in the diagnosis of the diseases of the ear. Some writers tell us that when the affection is confined to a single ear it announces some lesion of the peripheral extremity of the nerve; but that when both ears are attacked, and especially if some trouble of vision coexist, we may conclude that the central extremity of the nerve is implicated.

I can affirm that the occurrence of accidental sounds in the ear on one side as well as on both is observed in various diseases of the auditory apparatus, without any sign that the auditory nerve has suffered, either in its peripheral or central part. On the other hand, increased excitability of the auditory nerve almost always attacks both sides at the same time, (or at least very nearly so,) and gives rise to a remarkable change in the moral faculties, to depression of spirits, nervous headache, disinclination to study, and disturbance of the sleep, without any proof that the central part of the auditory nerve is affected. That such is the case is proved by the immediate benefit obtained in this form of disease by the use of gentle excitants, directed, in the form of vapour, towards the peripheral extremity of the nerve, through the Eustachian tube, the cavity of the tympanum, and the fenestra ovalis.

As to coincidence of this state with disordered vision, I am convinced that the latter depends on some change near the origin or traject of the optic nerves, which has no connection with any analogous change in corresponding portions of the auditory nerve.

It has been said that deafness connected with accidental sounds in the ear presents the following peculiarity:—Compression of the carotid arteries dissipates the false sounds and the deafness with them. I have never seen any example of this kind, nor is the assertion supported by a single case. Itard, indeed, cites one case, but it is far from conclusive. On compressing the carotids he succeeded in almost entirely removing the abnormal sounds; but he does not tell us that the accompanying deafness was *almost entirely* removed with them.

In the treatment of this affection local and general blood-letting is commonly employed, from the idea that it is connected with congestion; but we cannot admit the propriety of this mode of treatment if we reflect on the variety of diseases with which the existence of abnormal sounds in the ear is connected. In cases of nervous deafness blood-letting would be certainly injurious; it would have a very doubtful effect on the sounds, and would inevitably increase the deafness.—*Ann. de la Chir. Francaise. From Prov. Med. Journ.* Feb. 11, 1843.

### FRACTURE OF THE FEMUR IN A FEMALE 89 YEARS OLD.

BY WILLIAM F. HENDERSON, M. D.

Mrs. F., a meagre person, of temperate and regular habits, sustained a fracture of the left femur while in the dusk of the evening passing across the road and coming in contact with a horse and light baker's cart, the driver of which did not perceive her. On my seeing her a few minutes afterwards the nature of the injury was apparent. She was removed to her house, and the fracture ascertained to be oblique, and near the centre of the bone.

Apparatus not being at hand, she was placed in bed, the limb encased in pillows, so as to form a double inclined plane. Next morning, Sept. 11, the fractured portions were placed in exact apposition, after considerable difficulty in retaining them in situ. The limb was put on the double inclined plane formed by Macintyre's apparatus, with a long splint outside, another, shorter, inside the thigh, and each well padded. Thus was the extremity comfortably secured, and thus it remained, without a single untoward or bad occurrence, until

Oct. 26, the forty-fourth day, when the apparatus was removed, and the fractured part found to be firmly united, the tumor of callus being small, but sufficient to show the perfection of the cure, and allow motion of the limb in any direction. In a few days, by appropriate treatment, she was enabled to put her foot to the ground, and even to step out bearing the weight of the body. She has long ago been able to move without the aid of a stick: has been out of doors, and perfectly competent to take exercise on foot when the weather permits. No deformity existing in the parts.

This case is placed before the surgical world, particularly the junior portion, not to be too dissipirited in prognosticating osseous union in subjects far advanced in life; as in this instance, at first, it was pronounced not at all likely that any thing further than cartilaginous union would take place, and that, therefore, Mrs. F, would terminate her days as a cripple, from want of power in the system to unite the fractured bone. It is hoped its relation may call forth the remarks of any similar instances occurring in the practice of some of your numerous readers and correspondents.—*London Medical Gazette.* January 13, 1843.

---

### A TABULAR VIEW OF THE TREATMENT OF UTERINE HÆMORRHAGES.

BY WILLIAM CAMPS, M.D., EDIN.

### HÆMORRHAGE BEFORE PARTURITION.

A. *Slight Hæmorrhage.*—Horizontal position; perfect repose; cool air; cool acidulous fluids; low diet; bleeding, if there be symptoms of plethora. The bladder and rectum to be emptied.

B. *Severe Hæmorrhage.*—The same treatment as in A., except the bleeding (1.) At first, cold applications; then the ergot of rye, in doses of 12 grains, repeated three times, at intervals of ten minutes. And if these means are insufficient to arrest the hæmorrhage, plug the vagina, or in some especial cases rupture the membranes (2.)

### HÆMORRHAGE DURING PARTURITION.

*Slight Hæmorrhage.*

Os uteri not dilated, and not dilatable.

Membranes entire:—The same means as in A., except the bleeding, which is not indicated unless the symptoms of plethora are exceedingly pronounced.

Membranes ruptured:—As above.

Os uteri dilated.

Membranes entire:—The same means as in A.; then wait, or rupture the membranes (3.)

Membranes ruptured:—The same means as in A., and wait; if the pains are feeble or slow, give the ergot of rye (4.)

*Severe Hæmorrhage.*

Os uteri not dilated, and not dilatable.

Membranes entire:—The same treatment as in A., except the bleeding; then the refrigerants, as cold applications. In case these fail, and if the pains are feeble, the ergot of rye; then rupture the membranes. Lastly, if the state of the os uteri will not allow of turning, plug the vagina.

Membranes ruptured:—The same treatment as in A.; refrigerants; ergot of rye if the pains be slow or feeble. In case these fail, compression of the uterus; plug the vagina; introduce the hand, and deliver by turning (5.)

Os uteri dilated, or dilatable.

Membranes entire:—Rupture the membranes (6.;) and if this does not succeed, introduce the hand and turn, or apply the forceps.

Membranes ruptured:—Turning, if the head of the child has not descended into the cavity of the pelvis (7.) Apply the forceps if the head of the child have already descended into the cavity of the pelvis. Simple extraction if the child present by breech, knees, or feet.

(1.) *The ergot of rye* is employed here as hæmostatic; in the case supposed, there is not, at present, uterine pains; it is not probable that the employment of the ergot of rye should produce them, for hitherto this remedy appears to possess the property of increasing the uterine contractions only when they occur spontaneously, and not that of exciting them, when they do not already exist.

(2.) *The plug* will, in the first place, arrest the hæmorrhage; then, by the retention of the blood, and by its presence, it will irritate the *neck* and *orifice* of the uterus, and it will induce the expulsive contractions. These will dilate the *os uteri*, and this dilatation will allow either the rupture of the membranes, or the termination of the accouchement.

(3.) This rupture can have no inconvenience; it is a means of preventing the increase of the hæmorrhage. We may always dispense with it, and rest satisfied with waiting until the progress of the labour shall have arrested the hæmorrhage: the last method is after all perhaps the most prudent. A little *more* or a little *less* tendency to the increase of the hæmorrhage should determine the choice of the one or of the other method.

1st. *Wait*, if the hæmorrhage do not increase in any degree, and still more so if it diminish.

2d. *Rupture the membranes* if there be any tendency to increase of the hæmorrhage; this rupture will be profitably preceded or followed by the administration of some doses of the ergot of rye, if the uterine pains are feeble or slow.

(4.) It may be asked, if it would not be proper to terminate the accouchement in this case, since the parts concerned seem disposed to this termination. We think that if the fœtus presents in the usual manner, it is better not to be officious with the application of the *forceps* or *turning*, because the employment of these means would be more severe than the *slight* hæmorrhage which appears to demand their use.

(5.) This case is one of great delicacy; the application of the plug here requires great caution; for when the vagina is closed up, the blood may possibly accumulate in the cavity of the uterus, so that the patient may be lost, although no blood make its appearance externally; and the danger will be so much the greater, as the uterus shall have been more developed before the rupture of the membranes, and as the uterine contractions shall be more feeble. The application of the plug should be preferred, to the termination of the accouchement, only when the uterine contractions are sufficiently strong, and when, at the rupture of the membranes, only a very small quantity of the liquor amnii shall escape from the uterus. Again, the application of the plug demands great care of and attention to the patient, and should be followed by the application of a bandage round the abdomen sufficiently tight to resist the enlargement of the uterus. On the contrary, when the uterine contractions are feeble, when a large quantity of the liquor amnii shall have escaped at the moment of the rupture of the membranes, it will be necessary to overcome the resistance offered by the state of the os uteri, and terminate the accouchement by *turning*.

(6.) In this case we may be surprised at the advice to *rupture the membranes*, and *wait*, before adopting any other method, according as the retraction of the uterus may have, or may not have, arrested the hæmorrhage; it seems so important, both to the mother and to the infant, that the birth of the latter should be the result of the uterine contractions alone, rather than of manual interference, very often difficult, that it is very desirable to take the chance of a spontaneous accouchement at all times when there is the probability of obtaining it. It is to be understood that this recommendation to *wait*, is only admissible in case the uterine contractions are neither feeble nor slow.

(7.) We may certainly in this case have recourse to the *forceps*, but the employment of this instrument, when the head of the child is above the orifice, and not engaged in the cavity of the pelvis, frequently offers sufficient difficulty to render the delivery by turning preferable.

It will be seen that the indications are based on the slightness or severity of the hæmorrhage, and not on the circumstance of the insertion or non-insertion of the placenta on the neck of the uterus; not that this circumstance is a matter of indifference, for almost always the hæmorrhage produced by the detachment of the placenta inserted over the orifice of the uterus, is one of a severe and serious character, and demands immediately the employment of the means indicated for severe hæmorrhages. Sometimes the insertion of the placenta on the neck of the uterus occasions only a slight hæmorrhage: the author does not consider, then, as do the greater part of accoucheurs, that the insertion of the placenta on the neck of the uterus requires, in every case, the speedy termination of the accouchement, yet it may modify the employment of the means above indicated. For instance, if in a case of severe hæmorrhage the placenta covers entirely the os uteri, we cannot have recourse to the simple rupture of the membranes, as we could if such were not the case. If the os uteri be neither sufficiently *dilated*, or sufficiently *dilatable*, to allow the introduction of the hand, it will be necessary to employ the plug; if, on the contrary, it be sufficiently *dilated*, or sufficiently *dilatable*, it will be necessary to detach one of the sides of the placenta, in order to make a passage into the cavity of the uterus, and deliver by turning; but if a portion only of the placenta be inserted on the os uteri, leaving exposed a part of the membranes, we may proceed as if the placenta were not inserted at the os uteri. In no case does it seem advisable to make a passage through the placenta, as some accoucheurs have recommended. Lastly, if the placenta, pushed by the head or the breech of the fœtus, be entirely, or almost entirely detached, and has passed beyond the orifice of the uterus, we must extract it before the fœtus, for this organ is useless in these circumstances, and its presence in the vagina is an obstacle to the free exercise of the hand, or of instruments.—*Lond. Med. Gaz.* Jan. 13, 1843.

---

### CURE OF GLANDERS IN MAN.

A wagoner, nineteen years of age, entered the Hôpital de la Charité, in Paris, on the 18th of October, 1841. He complained of having felt ill for the week preceding, without being able to specify any particular seat of disease. Soon intense pains were felt in the ankle and knee-joints, and the muscles of the leg and thigh, though unattended with swelling or redness. His pulse became quick, thirst intense; headach and prostration. On the 25th of October pustules, filled with a purulent matter, appeared on the instep and upper surface of the three smaller toes of the left foot. These pustules broke, and cicatrisation was completed in a few days; but a diffused swelling now made its appearance in the anterior part of the superior third of the thigh, followed by two similar tumours, one on each leg. M. Mouneret, under whose care the patient was placed, now suspected the nature of the disease, and ascertained that one of the horses kept in the stable where the patient had been sleeping actually had the glanders. For the eight months ensuing tumours of a similar kind to the foregoing were successively and incessantly appearing on all parts of the upper and lower extremities, though they continued one after another to disperse, and nothing in the general condition of the patient, except his emaciation, gave cause for alarm; yet one curious collateral circumstance is stated. Early in December, 1841, a horse being inoculated with the matter from one of the abscesses, died in the course of five days, without, however, presenting during life any of the ordinary symptoms, or after death any of the usual appearances belonging to the disease.

The treatment of the patient was the same nearly throughout, consisting chiefly of decoction and extract of cinchona in large doses, with wine.

On the 5th of July, 1842, iodine with iodide of potassium was administered. This was followed by an attack of erysipelas in the left arm, and the iodine was suspended, to be resumed on the 17th. No new tumors had appeared during the previous two months; the cicatrisation of those still existing was soon afterwards completed, ane the patient was discharged perfectly cured on the 31st of July.

Andral, and other able pathologists who saw this case, were unanimous in pronouncing it a true instance of glanders. The journal from which we have extracted the above relation says,—"The case is unique. In all the instances of glanders in the human subject reported hitherto the disease has proved fatal."—*Lond. Lancet*, Feb. 18, 1843. *From Gaz. des Hop.*

---

### HUNTER'S OPERATION FOR ANEURISM.

Knowing that Mr. B. Phillips had been for some time collecting from English and foreign works the number of various surgical operations recorded, and their relative success, I applied to him to furnish me with the number he had been enabled to collect upon the subject of aneurism treated according to Hunter's method, and he has been so obliging as to furnish me with the following return. 389 cases of aneurism had been so treated, and the result 277 cures.

| | Cases. | Cures. |
|---|---|---|
| Subclavian . . . . | 80 | 46 |
| External iliac . . . | 79 | 62 |
| Carotid . . . . . | 74 | 59 |
| Femoral . . . . | 113 | 77 |
| Humoral . . . . | 30 | 24 |
| Various . . . . . | 13 | 9 |
| | 389 | 277 |

And when you consider that the operation, as an established one, has, of late years especially, been often performed without any record of it being published, you will perceive that I have not gone beyond the truth in asserting that it has conferred life upon hundreds.—*Arnott's Hunterian Oration.*

